# Deacetylation of Fungal Exopolysaccharide Mediates Adhesion and Biofilm Formation

**DOI:** 10.1128/mBio.00252-16

**Published:** 2016-04-05

**Authors:** Mark J. Lee, Alexander M. Geller, Natalie C. Bamford, Hong Liu, Fabrice N. Gravelat, Brendan D. Snarr, François Le Mauff, Joseé Chabot, Benjamin Ralph, Hanna Ostapska, Mélanie Lehoux, Robert P. Cerone, Stephanie D. Baptista, Evgeny Vinogradov, Jason E. Stajich, Scott G. Filler, P. Lynne Howell, Donald C. Sheppard

**Affiliations:** a Departments of Medicine and of Microbiology and Immunology, McGill University, Montréal, Canada; b Division of Infectious Diseases, Los Angeles Biomedical Medical Institute at Harbor-University of California, Los Angeles Medical Center, Torrance, California, USA; c Program in Molecular Structure & Function, The Hospital for Sick Children, Toronto, Canada; d Department of Biochemistry, Faculty of Medicine, University of Toronto, Toronto, Canada; e National Research Council, Ottawa, Canada; f Department of Plant Pathology and Microbiology, University of California, Riverside, Riverside, California, USA; g Infectious Diseases and Immunity in Global Health Program, Research Institute of the McGill University Health Centre, Montreal, Canada

## Abstract

The mold *Aspergillus fumigatus* causes invasive infection in immunocompromised patients. Recently, galactosaminogalactan (GAG), an exopolysaccharide composed of galactose and *N*-acetylgalactosamine (GalNAc), was identified as a virulence factor required for biofilm formation. The molecular mechanisms underlying GAG biosynthesis and GAG-mediated biofilm formation were unknown. We identified a cluster of five coregulated genes that were dysregulated in GAG-deficient mutants and whose gene products share functional similarity with proteins that mediate the synthesis of the bacterial biofilm exopolysaccharide poly-(β1-6)-*N*-acetyl-d-glucosamine (PNAG). Bioinformatic analyses suggested that the GAG cluster gene *agd3* encodes a protein containing a deacetylase domain. Because deacetylation of *N*-acetylglucosamine residues is critical for the function of PNAG, we investigated the role of GAG deacetylation in fungal biofilm formation. Agd3 was found to mediate deacetylation of GalNAc residues within GAG and render the polysaccharide polycationic. As with PNAG, deacetylation is required for the adherence of GAG to hyphae and for biofilm formation. Growth of the Δ*agd3* mutant in the presence of culture supernatants of the GAG-deficient Δ*uge3* mutant rescued the biofilm defect of the Δ*agd3* mutant and restored the adhesive properties of GAG, suggesting that deacetylation is an extracellular process. The GAG biosynthetic gene cluster is present in the genomes of members of the *Pezizomycotina* subphylum of the *Ascomycota* including a number of plant-pathogenic fungi and a single basidiomycete species, *Trichosporon asahii*, likely a result of recent horizontal gene transfer. The current study demonstrates that the production of cationic, deacetylated exopolysaccharides is a strategy used by both fungi and bacteria for biofilm formation.

## IMPORTANCE

This study sheds light on the biosynthetic pathways governing the synthesis of galactosaminogalactan (GAG), which plays a key role in *A. fumigatus* virulence and biofilm formation. We find that bacteria and fungi use similar strategies to synthesize adhesive biofilm exopolysaccharides. The presence of orthologs of the GAG biosynthetic gene clusters in multiple fungi suggests that this exopolysaccharide may also be important in the virulence of other fungal pathogens. Further, these studies establish a molecular mechanism of adhesion in which GAG interacts via charge-charge interactions to bind to both fungal hyphae and other substrates. Finally, the importance of deacetylation in the synthesis of functional GAG and the extracellular localization of this process suggest that inhibition of deacetylation may be an attractive target for the development of novel antifungal therapies.

## INTRODUCTION

*Aspergillus fumigatus* is an opportunistic mold that causes invasive infections in immunosuppressed patients. Despite antifungal treatment with the currently available antifungal agents, the mortality of invasive aspergillosis (IA) remains between 50 and 95% (1), highlighting the urgent need for new, effective therapeutic agents for this disease. Identifying and targeting virulence factors unique to this fungus is one approach to the development of novel treatments for invasive aspergillosis.

Galactosaminogalactan (GAG) is an exopolysaccharide recently identified in *A. fumigatus* that plays a number of roles in the pathogenesis of IA (2, 3). GAG is a linear heteropolymer composed of α-1,4-linked galactose and *N*-acetylgalactosamine (GalNAc) that is bound to the outer cell wall and found within the extracellular matrix of biofilms of *Aspergillus* species (4, 5). Cell wall-associated GAG mediates a number of virulence-associated traits, including adherence to host cells and other substrates, biofilm formation, masking of β-1,3-glucans from immune recognition, and resistance to neutrophil extracellular traps (2, 6). However, the biosynthetic pathways underlying GAG synthesis and the molecular mechanisms by which GAG mediates these virulence traits remain poorly understood.

**FIG 1:**
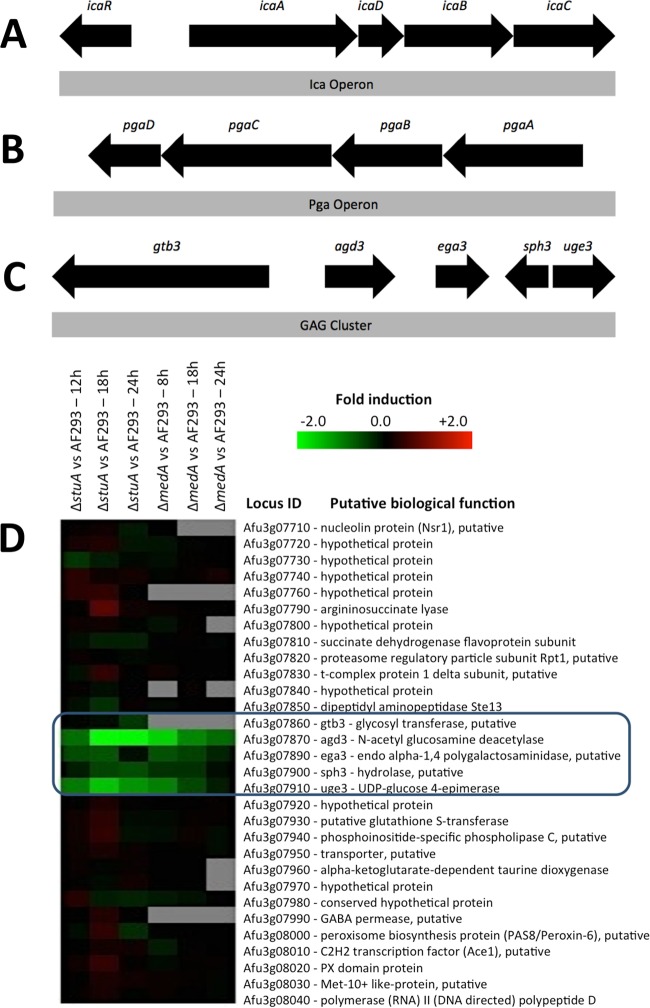
Bacterial polysaccharide biosynthetic operons and the putative GAG biosynthetic gene cluster. (A to C) Schematic of the *ica* operon responsible for the synthesis of polysaccharide intercellular adhesion (PIA) (A), *pga* operon responsible for the synthesis of PNAG [poly-(β1-6)-*N*-acetyl-d-glucosamine] (B), and the putative GAG gene cluster (C). (D) Heatmap showing differential gene expression of the *A. fumigatus* Δ*medA* and Δs*tuA* regulatory mutants compared to wild-type *A. fumigatus*, highlighting the coregulation of the genes in the GAG biosynthesis gene cluster. Fold induction is shown in red (upregulation), green (downregulation), black (no change), and gray (missing data point). Locus ID, locus identification.

**FIG 2:**
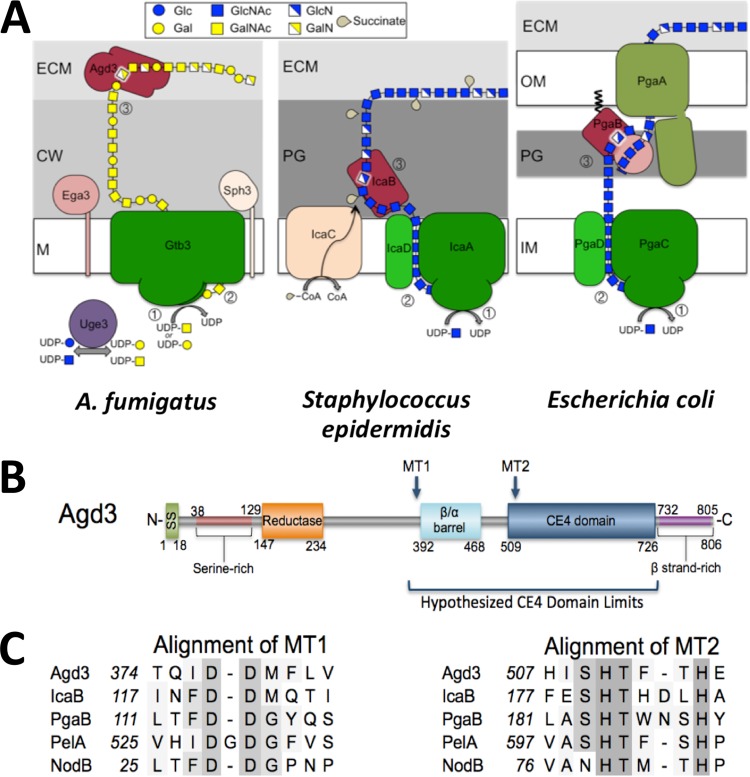
Comparative models and *in silico* analysis. (A) Comparative models of exopolysaccharide synthesis in bacteria and fungi. The numbered steps are as follows: (step 1) polymerization of sugar residues by the glycosyltransferases indicated in green (Gtb3, IcaA, and PgaC), (step 2) extrusion of the elongating polysaccharide from the cytosolic side to the extracellular side (or periplasm in Gram-negative bacteria) by the dual action of the glycosyltransferase (Gtb3, IcaA, and PgaC) and associated protein for the bacterial species (IcaD and PgaD), and (step 3) deacetylation of the *N*-acetylhexosamine unit of the nascent polysaccharide by the de-*N*-acetylase indicated in dark red (Agd3, IcaB, and PgaB). The extracellular matrix (ECM), cell wall (CW), plasma membrane (M), peptidoglycan (PG), outer membrane (OM), and inner membrane (IM) are shown. CoA, coenzyme A. (B) Predicted domains and conserved regions in the Agd3 protein. From the N terminus, these domains include signal peptide (SS), a serine-rich region, a glutamine amidotransferase domain (reductase), metal-coordinating linear motifs (MT1/MT2), β/α barrel, a carbohydrate esterase-4 like domain (CE 4), and a β-strand-rich region. (C) Multiple-sequence alignment showing conserved DXD/DD motif located within the MT1 and MT2 conserved sites. Sequences include *S. epidermidis* IcaB, *E. coli* PgaB, *P. aeruginosa* PelA, and *Sinorhizobium meliloti* NodB. Highly conserved and similar residues are highlighted in dark and light gray, respectively. Gaps introduced to maximize alignment are indicated by hyphens.

A number of pathogenic bacteria also produce exopolysaccharide composed of *N*-acetylhexosamines, most commonly β-1,6-linked *N*-acetylglucosamine (GlcNAc). This exopolysaccharide, known as polysaccharide intercellular adhesion (PIA) in *Staphylococcus aureus* (7) or poly-(β1-6)-*N*-acetyl-d-glucosamine (PNAG) in *Escherichia coli* (8), mediates many of the same virulence properties as GAG does, including adherence of the organism to both biotic and abiotic surfaces, resistance to neutrophil killing, and masking of pathogen-associated molecular patterns (PAMPs) (8–11). The mechanisms governing the syntheses of these bacterial exopolysaccharides have been well characterized and are mediated by the gene products of the *ica* or *pga* operons, respectively (Fig. 1A and B and Fig. 2). Briefly, PIA/PNAG is synthesized and transported across the membrane and extruded extracellularly by the action of a glycosyltransferase complex composed of either IcaA/D or PgaC/D, respectively. The nascent polysaccharide undergoes partial deacetylation of GlcNAc residues by IcaB/PgaB, resulting in interspersed glucosamine (GlcN) residues (11–13). In Gram-negative bacteria, the partially deacetylated polymer is then transported across the outer membrane by PgaA (14). Under acidic conditions, these GlcN residues are protonated, conferring a positive charge on the mature polysaccharide, and thereby mediating adherence to negatively charged surfaces including host cells and the organism itself, as well as enhancing resistance to cationic molecules such as aminoglycoside antibiotics and antimicrobial peptides (15). Loss of deacetylase activity results in the formation of a fully acetylated polysaccharide that cannot adhere to the organism or other substrates, and which is shed into the culture supernatant, rendering it nonfunctional (11, 16).

Although the type of hexosamine and linkages differ between PIA/PNAG and GAG, we hypothesized that the biosynthetic pathways for these glycans are similar and that partial deacetylation of GAG is also required for its function. Comparative transcriptome studies identified a cluster of genes in *A. fumigatus* that is similar in composition to the bacterial exopolysaccharide operons. This cluster encompasses the *uge3* gene, encoding a glucose 4-epimerase that is required for GAG biosynthesis (2, 17). Molecular and biochemical studies revealed that, as with bacterial exopolysaccharide, GAG is partially deacetylated. This deacetylation is mediated by Agd3, which is encoded by another gene within the GAG biosynthetic gene cluster. Extracellular Agd3 activity was required for binding of GAG to the hyphal cell wall, biofilm formation, masking of β-1,3-glucan, and full virulence. Bioinformatic analysis of fungal genomes revealed the presence of the GAG biosynthetic gene cluster within the genome of a number of other filamentous ascomycetes. The complete cluster and evidence for GAG production was also found within the basidiomycete *Trichosporon asahii*, likely as a consequence of a recent horizontal gene transfer event.

## RESULTS

### Identification of a gene cluster encoding putative GAG biosynthetic proteins.

Previously, comparative transcriptomic analysis of two transcription factor mutants, Δ*stuA* and Δ*medA* mutants, identified *uge3*, a gene encoding a bifunctional epimerase, as required for GAG synthesis (2, 17). A more detailed analysis of this data set revealed that the *uge3* gene is flanked by four other coregulated genes (Fig. 1C and D). These genes include genes predicted to encode a glycosyltransferase (*gtb3*), polysaccharide deacetylase (*agd3*), polysaccharide hydrolase (*ega3*), and spherulin-4-like protein (*sph3*). The predicted functions of these proteins suggest a model for exopolysaccharide synthesis analogous to the bacterial ICA/PGA systems (Fig. 2A). In this model, UDP-galactose and UDP-GalNAc are synthesized by the activity of the epimerase Uge3 and then polymerized and extruded from the cell by the putative transmembrane glycosyltransferase Gtb3. As with bacterial exopolysaccharide systems, the function of the putative glycosyl hydrolase within the fungal system is unclear, but a recent study from our group suggests that it may be required for polymer modification and remodeling (18). Importantly, the final step in this putative pathway is predicted to be the partial deacetylation of *N*-acetylgalactosamine residues within the nascent polymer by the deacetylase Agd3 to generate a polycationic glycan that adheres to the hyphal and other surfaces. To begin to validate this model, we therefore sought to determine the role of Agd3 in GAG deacetylation and function.

### Agd3 is predicted to contain a polysaccharide deacetylase domain.

**FIG 3:**
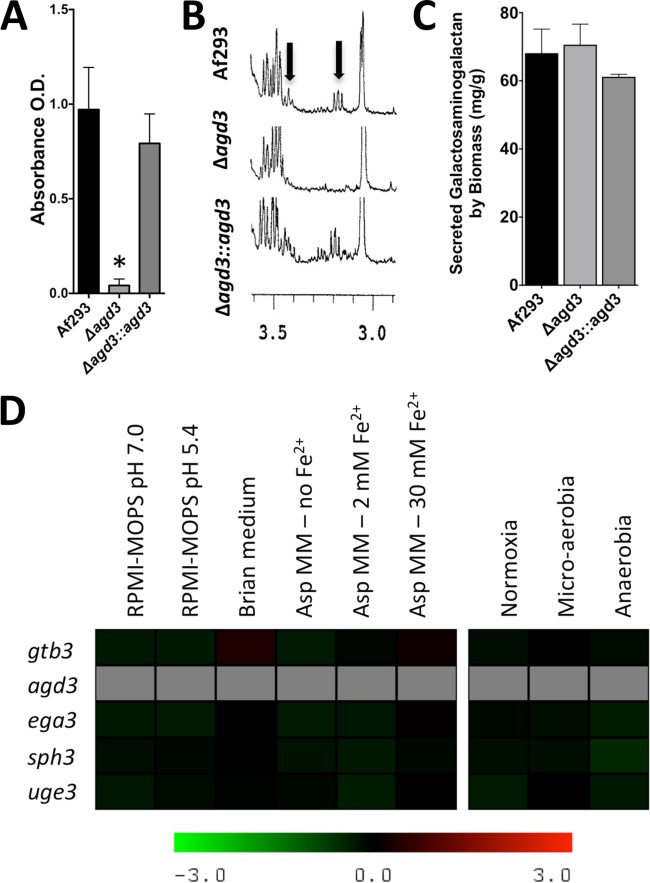
Deletion of *agd3* blocks deacetylation of GAG. (A) Detection of primary amine of purified GAG from the strains indicated in the figure as measured by evolution of colorimetric byproduct of the TNBS reaction. O.D., optical density. (B) ^1^H NMR analysis of purified GAG from the indicated strains. Black arrows indicate the detection of the hydrogen resonance peak originating from galactosamine. (C) Total secreted GAG production by the indicated strains. (D) Comparison of relative expression of GAG cluster genes between wild-type Af293 and Δ*agd3* mutant as measured against reference gene *tef1* under various growth conditions (RPMI 1640 supplemented with MOPS [RPMI-MOPS], Brian medium, *Aspergillus* minimal medium [AspMM]). For anaerobic growth, additional reference genes, *actin 1*, and *β-tubulin* were also used. For all graphs, data are represented as means plus standard errors of the means (SEM) (error bars). The values of the wild-type *A. fumigatus* Af293 and the Δ*agd3* mutant strain were significantly different (*P* < 0.05 by analysis of variance [ANOVA] with Tukey’s test for pairwise comparison) as indicated by the asterisk.

Bioinformatic analysis of Agd3 suggests that it is a multidomain extracellular protein including a predicted signal peptide with a cleavage site between Cys18 and Thr19 (19, 20), suggesting that Agd3 is secreted as an extracellular protein (Fig. 2B). Structure prediction server Phyre^2^ analysis of Agd3 predicted several distinct domains and extended regions of low complexity (21). The N-terminal region of the protein from residues 38 to 129 contains a serine-rich region that is predicted to be structurally disordered. When Agd3 lacking the signal peptide was analyzed using the structure prediction server Phyre^2^, the top hits (>95% confidence) for residues 509 to 726 were deacetylase domains of members of carbohydrate esterase family 4 (CE 4) (Fig. 3A). The CE 4 family contains a large number of metal-dependent polysaccharide deacetylases, which have been shown to remove *N*- or *O*-linked acetate groups from chitin, peptidoglycan, acetylxylan, and poly-β-1,6-*N*-acetylglucosamine (12, 22–24). CE 4 domains adopt a (β/α)_7_ fold with five canonical amino acid motifs that are important for active site formation and activity (12, 23). The structural prediction for Agd3 contains only a partial CE 4 fold starting at the second active site motif, which usually occurs at the end of the third β-strand of the barrel (Fig. 2B).

Using the whole protein, Phyre^2^ was unable to predict any function-associated motifs for residues 140 to 509 (25). However, when submitted to Phyre^2^ in isolation, residues 147 to 234 were predicted (86.2% confidence) to be similar to the flavodoxin-like fold present in *Thermotoga maritima* class I glutamine amidotransferase (GATase) (PDB code 1O1Y). Class I GATases are defined by a conserved Cys-His-Glu catalytic triad that is important for the reductase activity (25). These residues could not be identified in the Agd3 model, which contained only 88 of the 140 to 180 residues typically found in this fold. This domain is followed by a region of low structural complexity, as residues 243 to 300 were predicted to be largely disordered by both Phyre^2^ and GlobPlot. When residues 200 to 500 were submitted, a partial (β/α) barrel was identified between residues 392 and 468 with 75.3% confidence. This region may represent the first β/α repeats of the CE 4 domain.

The metal coordinating triad (Asp-His-His) is conserved in CE 4 enzymes and is localized to two of the five canonical linear motifs (MTs). The MT1, DD and DXD motifs contain the coordinating aspartic acid, as well as the catalytic base (Fig. 2C). In Agd3, MT1 is most likely D377 and D378, which are highly conserved in Agd3 orthologs. These residues are located outside the predicted CE 4 domain and slightly upstream of the partial (β/α) barrel motif. MT2 contains the metal coordinating histidines. In Agd3, H510 and H514, which are highly conserved and align with active site residues of known polysaccharide deacetylases using ClustalW, probably constitute MT2 (Fig. 2C). The linear distance between MT1 and MT2 is larger than that found in most CE 4 family members and is predicted to be a region of low structural complexity. Similar insertions between motifs are present in *Helicobacter pylori* PgdA and *Staphylococcus epidermidis* IcaB, where they are important for homomultimerization and membrane association, respectively (26, 27). Circular permutations of the CE 4 motifs have also been reported in multiple members (23, 26). Based on these findings, Agd3 has a unique CE 4 domain composition that may have prevented accurate modeling of the entire domain by Phyre^2^, making the exact domain boundaries unclear. Collectively, these *in silico* analyses suggest that Agd3 contains functional domains similar to known bacterial polysaccharide deacetylases, and therefore Agd3 may mediate the deacetylation of GAG.

### Deletion of *agd3* blocks deacetylation of GAG.

To determine whether GAG is partially deacetylated, the presence of primary amines in purified GAG was quantified by a chemical approach using trinitrobenzene sulfonate (TNBS) (28). This analysis identified that the presence of primary amines consistent with the presence of non-acetylated galactosamine (GalN) in GAG purified from wild-type *A. fumigatus* (Fig. 3A). The presence of GalN residues within purified GAG was also confirmed by nuclear magnetic resonance (NMR) analysis (Fig. 3B). Taken together, these data suggest that GAG undergoes partial deacetylation.

To determine whether Agd3 activity is required for GAG deacetylation, a Δ*agd3* deletion mutant strain was constructed. Deletion of *agd3* resulted in the production of GAG that was devoid of primary amines and lacked GalN as detected by NMR analysis (Fig. 3A and B). Deletion of *agd3* did not significantly affect the production of total secreted GAG (Fig. 3C) or affect the expression of other genes in the GAG biosynthetic gene cluster under all growth conditions tested (Fig. 3D). As previously reported for the deletion of *uge3*, loss of Agd3 did not affect susceptibility to antifungal agents or cell wall stressors, including caspofungin and nikkomycin (see Fig. S1 in the supplemental material). Complementation of the Δ*agd3* mutant with a wild-type allele of *agd3* restored deacetylation to wild-type levels. These data suggest that Agd3 is required for the deacetylation of GAG.

### Deletion of *agd3* is associated with the loss of adherence and changes in the cell wall morphology.

**FIG 4:**
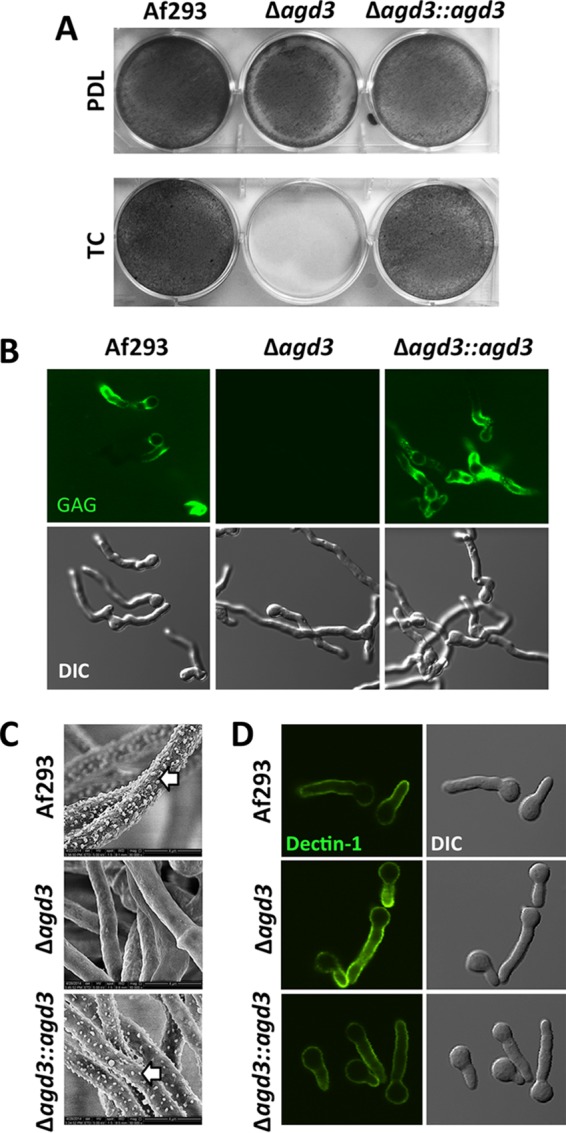
Deletion of *agd3* is associated with loss of adherence and changes in the cell wall. (A) Formation of adherent biofilms by the strains indicated in the figure on either positively charged (poly-d-lysine-treated [PDL]) or negatively charged (tissue culture-treated polystyrene [TC]) surfaces. Biofilms were washed and visualized by staining with crystal violet (gray). (B) Confocal microscopy images of hyphae stained with FITC-tagged soybean agglutinin lectin (top) and corresponding differential interference contrast (DIC) (bottom). (C) Scanning electron micrographs of hyphae grown for 24 h. The white arrows point to hyphal surface decorations associated with GAG production. (D) Confocal microscopy images of hyphae stained with Fc-dectin-1 detected by FITC-tagged Fc-receptor antibody (top) and corresponding DIC (bottom).

To examine the role of deacetylation and significance of the resulting cationic charge on the functional properties of GAG, the Δ*agd3* mutant was compared with the wild-type parent and *agd3* complemented strains for a variety of GAG-dependent phenotypes. Deletion of *agd3* was associated with a loss of adherence to negatively charged tissue culture-treated surfaces (Fig. 4A). However, consistent with the hypothesis that GAG-mediated adherence is dependent on charge-charge interactions, the Δ*agd3* mutant retained the ability to form wild-type biofilms on positively charged poly-d-lysine-coated plates (Fig. 4A). Thus, deacetylation of GAG mediates adherence to negatively charged surfaces, but not to positively charged surfaces.

The loss of Agd3 resulted in the loss of detectable cell wall-associated GAG as measured by direct immunofluorescence with the GalNAc-specific soybean agglutinin (SBA) lectin (Fig. 4B). These findings were also confirmed by scanning electron microscopy, which demonstrated a loss of the GAG-dependent cell wall decorations (Fig. 4C). Consistent with this loss of cell wall-associated GAG, hyphae of the Δ*agd3* mutant displayed increased exposure of β-1,3-glucan as detected by enhanced binding of recombinant Fc-dectin-1 by fluorescence microscopy (Fig. 4D; see Fig. S2 in the supplemental material). The phenotype of the Δ*agd3* mutant observed in these assays was indistinguishable from the GAG-deficient Δ*uge3* mutant (2). Collectively, these data suggest that Agd3-mediated deacetylation of GAG is required for adherence of the polysaccharide to the fungal cell wall and mediates adherence of hyphae to positively charged surfaces.

### De-*N*-acetylated GAG confers a positive charge on the hyphal surface.

**FIG 5:**
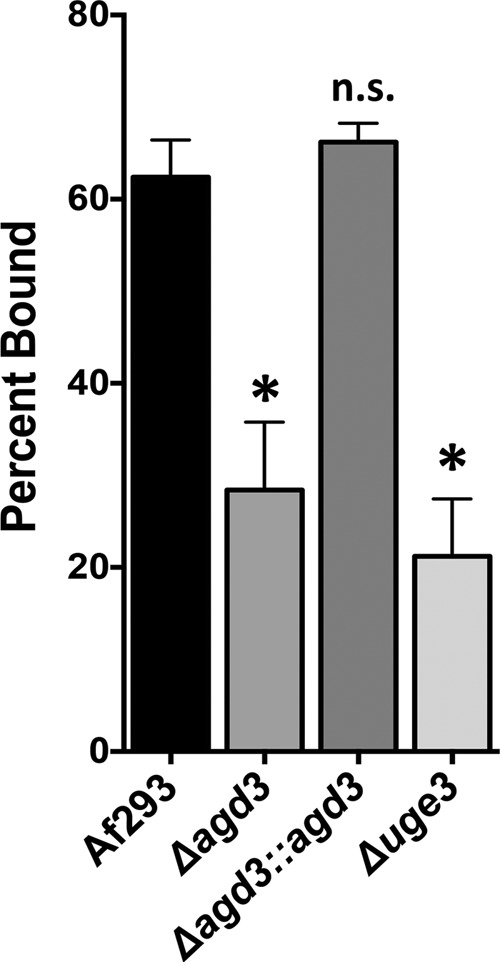
Agd3 activity augments the positive charge on the surfaces of hyphae. The graph shows the percentage of negatively charged Sephadex beads bound by hyphae of the indicated strains. Data are presented as means plus standard errors of the means (SEM) (error bars). The values for the indicated mutant strains were significantly different (*P* < 0.05 by ANOVA with Tukey’s test for pairwise comparison) from the value for wild-type *A. fumigatus* Af293 strain as indicated by the asterisk. The value for the Δ*agd3*::*agd3* strain was not significantly different (n.s.) from the value for the wild-type *A. fumigatus* Af293 strain.

To confirm that the presence of deacetylated GAG alters the surface charge of *A. fumigatus* hyphae, the ability of hyphae to bind negatively charged Sephadex beads was quantified (29). When coincubated with anionic Sephadex beads, there was a greater binding of negatively charged beads by wild-type *A. fumigatus* or by the *agd3* complemented strain than by the Δ*agd3* mutant (Fig. 5). Binding of anionic beads by hyphae of the Δ*agd3* mutant was similar to the binding of these beads by the completely GAG-deficient Δ*uge3* mutant. Thus, deacetylation is required to render GAG cationic and mediates adhesion to both the hyphal cell wall and other surfaces.

### Deletion of *agd3* attenuates virulence.

**FIG 6:**
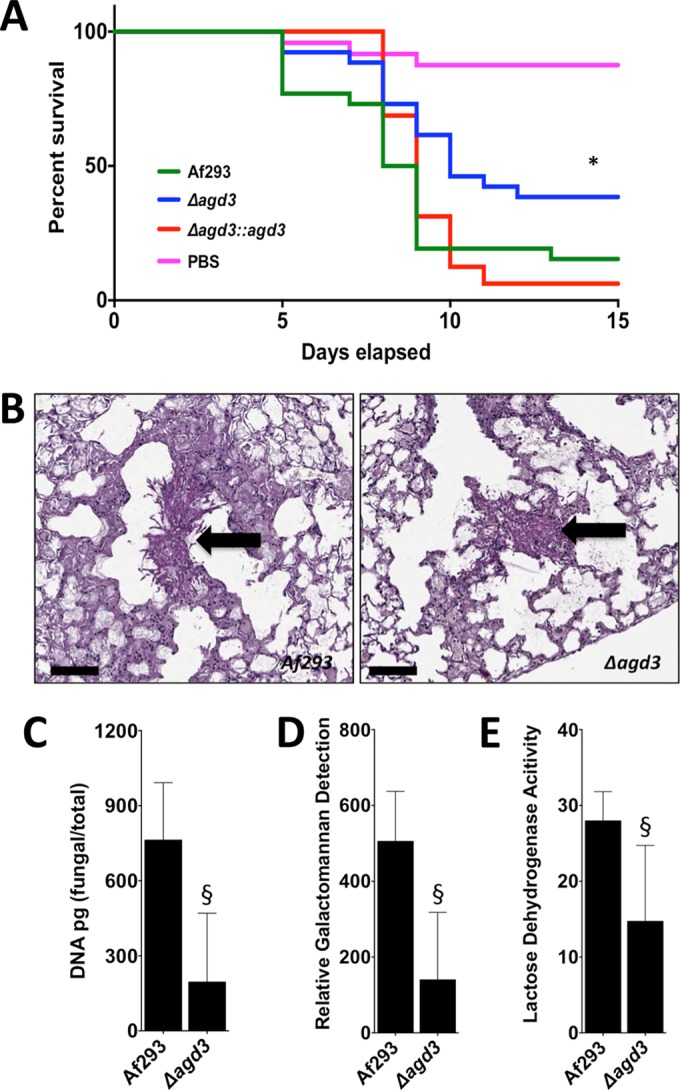
Agd3 is required for full virulence in a mouse model of invasive aspergillosis. (A) Survival of BALB/c mice treated with cortisone and cyclophosphamide and then infected with the indicated conidial strains. Graphs are the combined results of two independent experiments with 26 mice per group for all groups of mice infected with fungal strains and 24 mice in the PBS sham infection group. There was a significant difference in the survival of mice infected with wild-type Af293 or Δ*agd3*::*agd3* strain compared to those infected with the Δ*agd3* strain as determined by the Mantel-Cox log rank test with pairwise comparison applying Bonferroni’s correction as indicated by the asterisk. (B) Pulmonary histopathology sections from BALB/c mice infected with indicated strains and stained with PAS for visualization of fungal elements. Black arrows indicate fungal elements found within pulmonary lesions. Bars, 100 µm. (C) Pulmonary fungal burden of mice infected with the indicated strains, as measured by quantitative PCR. There were 8 mice in each group. (D) Pulmonary fungal burden of mice infected with the indicated strains, as measured by determination of pulmonary galactomannan content. There were 8 mice in each group. (E) Pulmonary injury as measured by lactose dehydrogenase activity in the bronchoalveolar lavage fluid of mice infected with the indicated strains. There were 8 mice in each group. Values are medians plus interquartile ranged (error bars). There was a significant difference in either the fungal burden or lung injury in mice infected with wild-type Af293 strain and those infected with the Δ*agd3* mutant strain as determined by the Mann-Whitney test as indicated by the § symbol.

To confirm that deacetylation of GAG plays a role in pathogenesis, the virulence of the Δ*agd3* mutant was compared to the wild-type Af293 strain and the *agd3* complemented strain in a leukopenic murine model of invasive aspergillosis (Fig. 6A) (2, 30). Consistent with the *in vitro* findings of impaired GAG function, the Δ*agd3* mutant was hypovirulent compared to wild-type *A. fumigatus* or the *agd3* complemented strain. Histopathologic examination of the lungs of mice infected with the Δ*agd3* mutant revealed no difference in the appearance of fungal lesions in mice infected with the wild-type or Δ*agd3* mutant strains (Fig. 6B). However, determination of fungal burden by quantitative PCR (Fig. 6C) or pulmonary galactomannan content (Fig. 6D) revealed a lower fungal burden in the lungs of mice infected with the Δ*agd3* mutant than in mice infected with the wild-type parent strain, as was previously reported with the Δ*uge3* mutant strain. Consistent with the lower fungal burden observed in mice infected with the Δ*agd3* mutant, lower pulmonary injury was observed in these animals as measured by lactose dehydrogenase activity in bronchoalveolar lavage fluid (Fig. 6E). Thus, deacetylation of GAG is required for full virulence of *A. fumigatus*.

### Restoration of biofilm formation by coculture of biofilm-deficient strains.

**FIG 7:**
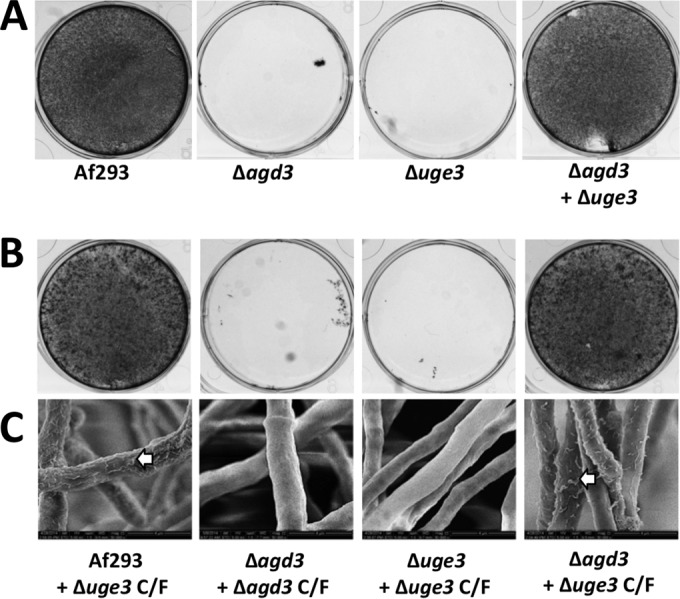
Culture filtrates from the Δ*uge3* mutant complement the defects in adherence and cell wall morphology of Δ*agd3* mutant. (A) Biofilm formation by the indicated strains grown alone or in coculture. After the biofilm was washed, adherent biofilm was visualized by crystal violet staining. (B) Biofilm formation by the indicated strains grown in the presence of culture filtrates (C/F) from the Δ*uge3* or Δ*agd3* mutant. After the biofilm was washed, adherent biofilm was visualized by crystal violet staining. (C) Scanning electron microscopy visualization of cell wall morphology of the indicated strains grown in the presence of culture filtrates from the Δ*uge3* mutant. The white arrows indicate cell wall decorations associated with cell wall-bound GAG.

Since the Δ*agd3* mutant produces fully acetylated GAG but not Agd3, and the Δ*uge3* mutant produces Agd3 but not GAG, we hypothesized that these strains could complement one another to produce deacetylated GAG and form biofilms. As predicted, coculture of the Δ*uge3* and Δ*agd3* mutants resulted in the formation of biofilms that were indistinguishable from those produced by wild-type *A. fumigatus* (Fig. 7A). Further, growth of the Δ*agd3* mutant in the presence of culture filtrates from the Δ*uge3* mutant resulted in the formation of adherent biofilms (Fig. 7B) and restored the presence of GAG-associated cell wall decorations as detected by scanning electron microscopy (Fig. 7C). Growth of the Δ*agd3* or Δ*uge3* mutant in the presence of their own respective culture filtrates had no effects on adherence (Fig. 7B) or hyphal morphology (Fig. 7C).

To extend these findings, we also examined the ability of cell-free culture filtrates from both Δ*agd3* and Δ*uge3* mutants to generate deacetylated GAG *in vitro* in the absence of *A. fumigatus* hyphae. Culture supernatants of each mutant or a 1:1 mixture of both culture supernatants were incubated in anionic polystyrene enzyme immunoassay (EIA) plates to capture adherent, deacetylated GAG. Adherent, deacetylated GAG was then quantified using an anti-GAG antibody (3). A 1:1 mixture of culture supernatants of the Δ*agd3* and Δ*uge3* mutants resulted in detection of adherent GAG at a level similar to that recovered from culture supernatants of wild-type *A. fumigatus* (see Fig. S3 in the supplemental material), while no adherent GAG was detected in culture filtrates of the Δ*agd3* mutant or Δ*uge3* mutant alone. Collectively, these results support the proposed model of GAG biosynthesis in which GAG undergoes deacetylation by extracellular Agd3.

### Agd3 is localized on the hyphal surface.

**FIG 8:**
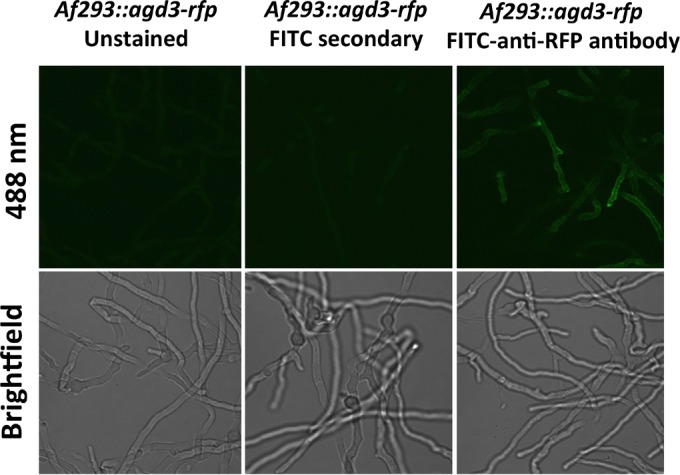
Agd3 localizes to the surfaces of hyphae. Hyphae expressing an Agd3-RFP fusion protein are visualized by confocal microscopy and indirect immunofluorescence.

The results of our biochemical and complementation studies suggest that Agd3-mediated deacetylation is an extracellular process. Consistent with these findings, a recent study identified Agd3 within the secretome of *A. fumigatus* (31). To determine whether Agd3 is also present within the cell wall, a red fluorescent protein (RFP)-tagged *agd3* allele was expressed under the native *agd3* promoter in *A. fumigatus* Af293. In this strain, while low levels of red fluorescence in the cell wall were observed, the signal was obscured by significant red autofluorescence of hyphae. To enhance visualization of RFP-tagged Agd3 (Agd3-RFP), indirect immunofluorescence using a fluorescein isothiocyanate (FITC)-tagged anti-RFP antibody was performed. Using this technique, low levels of Agd3-RFP were observed on the surfaces of hyphae (Fig. 8). Western blot analysis of culture supernatants and fungal biomass confirmed that although Agd3-RFP was found both in the culture supernatant and the fungal biomass, the majority of Agd3-RFP was associated with hyphae (see Fig. S4 in the supplemental material). Interestingly, from the fungal biomass, four discrete bands were detected using anti-RFP antibodies, while in the secreted fraction, only a single band was observed. The same pattern of bands was detected in multiple independent experiments. Liquid chromatography-mass spectrometry (LC-MS) analysis of all five of these bands confirmed that these bands contained Agd3-RFP. Further, the relative abundance of individual peptides did not vary significantly between these bands, suggesting that the differences in band migration are more likely due to differences in glycosylation rather than proteolytic cleavage.

### The GAG biosynthesis gene cluster is widespread in *Ascomycetes* but present only in *Trichosporon asahii* among *Basidiomycetes*.

**FIG 9:**
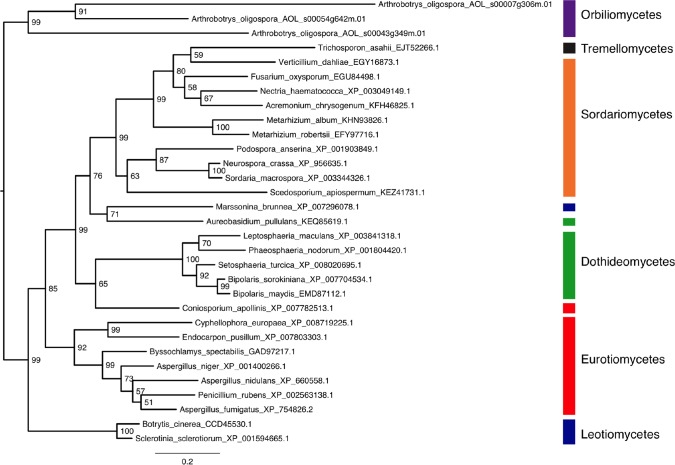
Agd3 orthologs are found within a wide range of *Ascomyces* species. A gene tree is shown categorized by taxonomic class of Agd3 orthologs found in the 28 fungal species that possess the GAG gene cluster. The gene tree was built by aligning and trimming Agd3 ortholog sequences, followed by maximum likelihood phylogenetic analysis with 100 bootstrap replicates. The outgroup was rooted with sequences of *Arthrobotrys* species. The scale bar is the genetic distance representing amino acid substitutions per site.

Using an *in silico* approach, the GAG gene cluster sequences from *A. fumigatus* were queried against more than 250 publically available fungal genomes at NCBI Genomes and FungiDB (32). Orthologous genes of the GAG gene cluster present at the same locus were found in 28 fungal species. In the majority of these species, synteny of the cluster genes was observed, suggesting a common origin for this cluster in these fungal species (see Fig. S5 in the supplemental material). All of the species containing the GAG gene cluster belong to the *Pezizomycotina* subphylum of the *Ascomycota* with the exception of one member of the phylum *Basidiomycota*, *Trichosporon asahii* (Fig. 9). More than half of the fungal species that possess the GAG gene cluster have been described as pathogenic species (see Table S1 in the supplemental material). Of these species, 13 are plant pathogens, while only 3 are known pathogens to insects and animals, suggesting a possible role for GAG in the pathogenesis of plant fungal infections.

To elucidate the evolutionary relationship among these gene cluster-containing species, and specifically of Agd3 orthologs, we constructed a gene tree by aligning the amino acid sequences of Agd3 orthologs from all 28 gene cluster-containing species (Fig. 9) and constructed a maximum likelihood tree rooted by the copies from the early branching ascomycete and nematode-trapping fungus *Arthrobotrys oligospora*. While the relationship among the orthologs of Agd3 largely mirrors the evolutionary relationship of the respective species as established by the fungal tree of life (33, 34), we found that the *T. asahii* Agd3 ortholog was nested in the tree within members of the class *Sordariomycetes* (Fig. 9), suggesting that a recent horizontal gene transfer event may have occurred. Consistent with this hypothesis, a predicted mutator-like element (MULE) domain-containing protein was found within the GAG gene cluster of *T. asahii* (see Fig. S5 in the supplemental material). The MULE domain-containing proteins are class II DNA transposases (35) and are found in fungal species (36) and are commonly found in eukaryotes (37). Analysis of the sequences flanking the cluster did not reveal an identifiable tandem inverted repeat at the borders of the GAG synthetic cluster in *T. asahii* or any other mobile element signatures. It therefore seems most likely that the transposable element is no longer active and that only the transposase domain remains, while other features of the mobile element have degraded.

**FIG 10:**
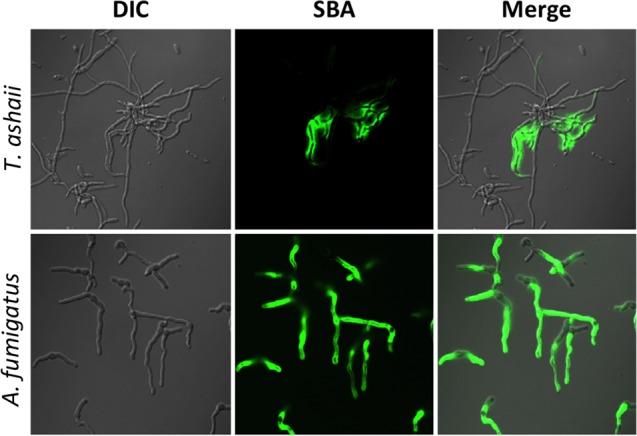
*Trichosporon asahii* produces a GAG-like exopolysaccharide. Hyphae of *T. asahii* were stained with FITC-tagged soybean agglutinin (SBA) lectin binding for the detection of GalNAc-rich exopolysaccharide.

To ascertain whether the GAG cluster was functional in *T. asahii*, mature hyphae of this organism were stained with fluorescein-tagged SBA lectin to detect the production of GAG. Lectin staining suggests that *T. asahii* secretes GAG into the extracellular matrix (Fig. 10). GAG produced by *T. asahii* was less uniformly bound to the cell wall of hyphae compared with *A. fumigatus* and seemed to form a matrix between hyphae.

## DISCUSSION

In the present study, we report a cluster of coregulated genes on chromosome 3 of *A. fumigatus* with composition and functions similar to those of bacterial operons encoding proteins required for exopolysaccharide synthesis. Characterization of one of the enzymes in this cluster demonstrates that, as with bacterial exopolysaccharide, deacetylation of GAG is required for GAG to adhere both to substrates and to the organism itself, and for full virulence. Further, these studies suggest that GAG-mediated adherence is largely a consequence of charge-charge interactions between the polycationic polysaccharide and negatively charged surfaces.

The results of this study illustrate important similarities between the Agd3 deacetylase and other multidomain CE 4 family members from bacteria. In *S. epidermis*, deacetylation of PIA by the CE 4 domain-containing deacetylase IcaB is required to confer a net positive charge to PIA and retain the polysaccharide on the bacterial cell wall (38). Similarly, PelA, a deacetylase in the *Pseudomonas aeruginosa* Pel exopolysaccharide biosynthetic operon, has also been reported to be required for the production of adhesive polysaccharide and biofilm formation in this organism (26). As with Agd3, PelA contains a small reductase fold and CE 4 deacetylase domain separated by a region of predicted low complexity, as well as a β-sheet-rich region at the C terminus. While the role of the predicted reductase and C-terminal domains in PelA are unknown, the similarities between the domain arrangements within PelA and Agd3 suggest they likely mediate similar functions in the synthesis of Pel and GAG, respectively. PgaB, from *E. coli*, requires an adjacent domain, located C terminally to the CE 4 domain, for the deacetylation of substrate (20). While there are domains other than CE 4 predicted in Agd3, their specific role in deacetylation is currently unknown. Finally, a protein containing the CE 4 domain, PssB, is required for the production of cell surface-bound extracellular polysaccharide in *Listeria monocytogenes* (39). Loss of PssB results in impaired bacterial cell aggregation and biofilm formation. Thus, postsynthesis modification of extracellular polysaccharides by deacetylation of hexosamine sugars seems to be a common theme used by diverse microorganisms in the formation of biofilms (40).

Although these polysaccharide systems display many similarities, several lines of evidence suggest that these biosynthetic pathways are the product of convergent, rather than divergent, evolution from a common ancestor of both fungi and bacteria. First, the polysaccharide composition and linkages of the exopolysaccharide product of these systems differ markedly between organisms. Although the composition of Pel is unknown, PIA/PNAG is composed of β-1,6-linked GlcNAc residues; the exopolysaccharide of *L. monocytogenes* is composed of a β-1,4-linked *N*-acetylmannosamine chain decorated with a terminal α-1,6-linked galactose; and GAG is a heteropolymer of α-1,4-linked galactose and GalNAc. Further, there is little sequence similarity between these bacterial and fungal enzymes beyond the conserved enzymatic domains themselves.

While it is possible that the GAG gene cluster arose *de novo* in fungi, it is also possible that these genes were acquired by horizontal gene transfer by a common ancestor of the *Pezizomycotina* that may have originated in bacteria or another organism. The clustered consecutive organization of the GAG biosynthetic genes is reminiscent of a bacterial operon and is relatively unusual in fungi. Fungal gene clusters have been best described as encoding enzymes required for the biosynthesis of secondary metabolites. Horizontal gene transfer of these secondary metabolite clusters has been suggested as a mechanism for their acquisition (41). The observations that *T. asahii* is the only basidiomycete found to contain the GAG gene cluster and that the *T. asahii* Agd3 ortholog nests with strong bootstrap support within the *Soridariomycetes* sequences in the gene tree are very strong evidence for a horizontal gene transfer event from an ascomycete species belonging to the *Sordariomycetes* class into *T. asahii* or its ancestor. Furthermore, the homology of *T. asahii* Agd3 and overall synteny of the cluster genes with other ascomycetes strongly suggest that the entire cluster was acquired in *T. asahii* or its parental linages as a single horizontal transfer event. Although sequence analysis did not reveal any clear evidence of a specific mechanism and integration of the gene transfer, it is possible that it was mediated by a MULE domain-containing transposase, which is found near the boundary of the cluster in *T. asahii*. As genomes of more fungal species are made available and queried, a better picture of the timing of this exchange and overall evolution of this gene cluster will likely become clearer.

Interestingly, many of the species containing the GAG gene cluster are known plant pathogens, while no primary animal-associated fungi, such as the dermatophytes or *Onygenales* fungi, contain a homolog of the gene. Whether the ability to produce GAG confers an advantage to colonize and invade plant hosts and whether this selection pressure resulted in the convergent evolution of cationic polysaccharides in microorganisms remains to be determined. The observation that bacterial and fungal pathogens have developed similar strategies to produce cationic exopolysaccharide might suggest that these polymers facilitate pathogenicity. However, as humans are dead-end hosts for *Aspergillus* species, it is likely that the pressures responsible for the development of GAG were found in the soil or other natural environment. A shared requirement for adherence to substrates and resistance to environmental stresses through the production of an adherent layer of exopolysaccharide may underlie the development of exopolysaccharide synthesis by all of these microorganisms. Future studies examining the role of exopolysaccharide in fungal and bacterial growth in nonhuman environments may shed light on the factors leading to the evolution of these glycans in these varied microorganisms.

Deacetylation of GalNAc by Agd3 is required for *A. fumigatus* to produce cell wall-bound GAG and for mediating the phenotypes that have been associated with cell wall-associated GAG. Further, the mixed mutant biofilm studies reported here would suggest that, as with Gram-positive bacterial exopolysaccharide, deacetylation occurs in the extracellular space. This observation suggests that inhibition of GAG deacetylation may represent an attractive antifungal target since intracellular penetration of a candidate deacetylase inhibitor would not be required for activity. Deacetylation of GalNAc within human cells has not been reported, suggesting that antifungal specificity may be achievable. As loss of GAG deacetylation has no direct effect on fungal viability, such a strategy would be best paired with a conventional antifungal agent.

Although a previous report did not identify the presence of deacetylated GalNAc within secreted GAG from *A. fumigatus* (3), compositional analysis of GAG from *Aspergillus niger* and *Aspergillus parasiticus* has reported the presence of GalN in preparations from these species (42, 43). Because deacetylated GAG adheres avidly to glass and plastics, it is possible that during the purification of *A. fumigatus* GAG, the preparation was enriched for fully acetylated, nonadherent polysaccharide. Alternately, it is possible that deacetylation varies under different growth conditions as reported in other species (44–46). Further experiments are required to test these possibilities.

In conclusion, we report that deacetylation of GAG by Agd3 is essential for the function of this exopolysaccharide, including adherence of GAG to the fungal cell wall and other substrates. These experiments shed light on the molecular mechanisms underlying GAG-mediated adhesion and draw important parallels between this glycan and bacterial exopolysaccharides. Finally, these data suggest the exciting possibility that targeting deacetylation of GAG may represent a promising antifungal strategy.

## MATERIALS AND METHODS

### Fungal strains and growth conditions.

*Aspergillus fumigatus* strain Af293 (a generous gift from P. Magee, University of Minnesota, St. Paul, MN) was used as the parent strain for all genetic manipulations and experimental controls. Unless otherwise noted, all *A. fumigatus* strains were maintained on yeast extract-peptone-dextrose (YPD) agar (Fisher Scientific) at 37°C. For growth in liquid culture, Brian medium (3) or phenol-free RPMI 1640 (Wisent) was used as indicated in the figures at 37°C and 5% CO_2_ incubation, unless otherwise noted. A clinical isolate of *Trichosporon asahii* was obtained from the McGill University Health Centre, Montreal, Québec, Canada, and maintained on potato-dextrose medium at 30°C.

### Molecular and genetic manipulations.

A split marker, double homologous recombination approach was used to generate the Δ*agd3* mutant (47). Sequences flanking the open reading frame (ORF) of the *agd3* gene were amplified from the genomic DNA using PCR (see Table S2 in the supplemental material). The resulting PCR fragments were then cloned into the entry vector pENTR-D-TOPO (Gateway, Inc.) and then recombined with previously described destination plasmids, pHY and pYG, containing the hygromycin split marker resistance cassettes (2). Target DNA was amplified by PCR from the recombined plasmids and then used for transformation of *A. fumigatus* as previously described (47).

To generate the Δ*agd3*::*agd3* complemented strain, the *agd3* open reading frame and flanking sequences (680 bp downstream and 560 bp upstream) were amplified by PCR using the Sgf-compl-fow (compl stands for complemented, and fow stands for forward) and Asc-compl-rev (rev stands for reverse) primers, respectively (see Table S2 in the supplemental material). The resulting PCR fragment was cloned into pTAPA, a newly designed destination plasmid containing the phleomycin selection marker. The resulting pTAPA::*agd3* plasmid contained the following sequence: P*agd3*::ORF*agd3*::T*cyc1*::ORF*ble*::P*thiA*::T*trpC*. Two fragments for transformation were amplified by PCR from the pTAPA::*agd3* plasmid using Sgf-compl-fow and LE4 primers and BL4 and Asc-compl-rev primers. Phleomycin-resistant transformants were selected, and correct integration at the native locus was verified by genomic PCR. Restoration of *agd3* mRNA expression was confirmed by real-time reverse transcription-PCR (RT-PCR) (Table S2).

To generate the *agd3*::*rfp* mutant, the ORF of *agd3* was amplified using primers AgeI-agd-end-fow and EcoRV-agd-end-rev (see Table S2 in the supplemental material), cloned upstream of the *rfp* ORF in the pRFPγ plasmid (17), and digested by AgeI and EcoRV, which excised the *gpdA* promoter region on the plasmid. Amplification of the linearized plasmid produced the (*agd3*-end-ORF)::(RFP)::(TtrpC)::(3′-half of HYG cassette) PCR fragment. From the *agd3* terminator on the pRFPγ plasmid, a 735-bp PCR product was made, using BsrGI-agd-Tr-fow and SacI-agd-TR-rev. This product was cloned upstream of the hygromycin (HYG) cassette in the pAN7.1 plasmid (2), digested by BsrGI and SacI, resulting in an amplified PCR fragment (5′-half of HYG cassette)::(*agd3*-Tr). *A. fumigatus* Af293 was then transformed with this construct (47). All integrations were of *agd3*, and integration of the hygromycin resistance was verified by genomic PCR confirmed using real-time RT-PCR (see Table S2 for relevant primers) (2).

### Gene expression studies.

Expression of the galactosaminogalactan (GAG) cluster genes AFUA_3G07910 (*uge3*), AFUA_3G07900 (*sph3*), AFUA_3G07890 (*ega3*), and AFUA_3G07860 (*gtb3*) in the Δ*agd3* mutant was compared to the wild-type Af293. The fungal strains indicated in the figures were grown for 18 h at 37°C under various growth conditions including phenol-free RPMI 1640 (Wisent) buffered with morpholinepropanesulfonic acid (MOPS) (Bioshop, Inc.) at pH 5.4 and pH 7.0, Brian medium (3), or *Aspergillus* medium (2) with iron supplementation of 0, 2, or 30 mM. For anaerobic (AnaeroPack; Mitsubishi Gas Chemical, Inc.) or microaerophilic (MicroAeroPack; Mitsubishi Gas Chemical, Inc.) conditions, fungi were grown in phenol-free RPMI 1640 (Wisent) buffered with MOPS (Bioshop, Inc.) at pH 7.0 in anaerobic chambers with the appropriate gas packs. Mycelia were collected, RNA was extracted, and quantitative PCR (qPCR) was performed and analyzed as previously described (see Table S2 in the supplemental material) (2).

### Bioinformatic analyses.

For homology structural analysis, the amino acid sequence of Agd3 from *Aspergillus fumigatus* was obtained from the Aspergillus Genome Database (19) and analyzed using a number of different web-based servers, including Phyre^2^, SignalP v3.0, GlobPlot, NetOGlyc 4.0, and SMART (20, 21, 48). The full-length sequence of Agd3 was initially used for the bioinformatic analysis. As analysis of full-length Agd3 using Phyre^2^ failed to predict a structural model for residues 140 to 500, these residues were submitted to the server with different boundaries including residues 140 to 250, 141 to 400, 200 to 500, 300 to 806, and 350 to 733. Alignments were performed using ClustalW2 and Phyre^2^ (20, 22).

To assess the prevalence of the GAG gene cluster across the fungal kingdom, amino acid sequences of each gene in the cluster in *A. fumigatus* was queried with FastA to search the predicted proteomes of publically available taxa in the *Ascomycetes* and *Basidiomycetes* (49). Species having a cluster containing a predicted glycosyltransferase, epimerase, deacetylase, and either the hydrolase or spherulin-like protein were selected. To determine the phylogenetic relationship of Agd3 among these species, a phylogenetic tree was constructed by first performing multiple alignment using T-Coffee (50), trimming the alignment with trimAl (51), followed by phylogenetic analysis using RAxML (v 8.1.1) with 100 bootstrap replicates and PROTGAMMA matrix with automatic model selection (52). The resultant tree was visualized with FigTree, and colors were added by using Adobe Illustrator (Adobe Systems Inc., Palo Alto, CA).

### Polysaccharide analysis.

GAG was extracted by ethanol precipitation and purified as previously described (17). For chemical analysis to quantify the presence of primary amines in GAG, colorimetric detection using trinitrobenzene sulfonate (TNBS) (Fisher Scientific, Inc.) was performed following the manufacturer’s instructions. For nuclear magnetic resonance (NMR) detection of the primary amine group, dried GAG samples were reconstituted in deuterated water, and cold deuterium chloride was added to dissolve the samples. ^1^H heteronuclear multiple quantum correlation (HMQC) NMR experiments were conducted on a Varian INOVA 500-MHz spectrometer with a 3-mm gradient probe. Assignment of spectra was performed using Bruker Topspin version 3.1 program for spectrum visualization.

### Hyphal surfa ce characterization.

For biofilm adherence assays, fungi were grown for 24 h in Brian medium on tissue culture-treated six-well polystyrene plates. Nonadherent fungi were removed by vigorous agitation and washing. Adherent biofilms were visualized by crystal violet staining (53). Cell wall topography was examined using scanning electron microscopy as previously described (2). For soybean agglutinin (SBA) or recombinant Fc-dectin-1 binding to the surface of hyphae, strains were grown for 7 to 9 h on poly-d-lysine-coated coverslips (BD Biosciences, Inc.), then fixed, and stained with either fluorescein-tagged SBA or recombinant Fc-dectin-1 (2). For all microscopy experimentation, strains were grown in phenol red-free RPMI 1640. The susceptibility of strains to cell wall-active antifungals was determined as previously described (2). Bright-field images of mycelia were captured at a magnification of ×200 (Infinity 2 camera; Lumenera Inc.).

Difference in hyphal surface charge was determined by hyphal binding to negatively charged CM Sephadex beads (Sigma Aldrich, Inc.). The fungal strains indicated in the figures were grown in RPMI 1640 in 50-ml centrifuge tubes at 37°C and 5% CO_2_ for 18 to 24 h. Reconstituted sterile beads were added to the tubes containing pregrown fungi, andthe tubes were then shaken for 10 min at 200 rpm. Supernatant samples were taken, and the number of unbound beads was counted by using a light microscope.

### Agd3 localization studies.

To determine the localization of Agd3 by fluorescence microscopy, the Af293 *agd3-rfp* mutant was grown on glass coverslips in RPMI 1640 for 9 to 12 h at 37°C and 5% CO_2_, fixed with 4% paraformaldehyde, stained with anti-red fluorescent protein (anti-RFP) IgG antibody (Abcam, Inc.), and detected with fluorescein-tagged anti-IgG antibody (Abcam, Inc.). The cells were then mounted on microscope slides and imaged by confocal microscopy as previously described (2). For RFP-tagged Agd3 (Agd3-RFP) immunoblotting, mycelia of the wild-type and Agd3-RFP strains were grown at 37°C for 22 to 24 h under shaking conditions, and culture supernatants were recovered by filtration. The biomass was crushed under liquid nitrogen, homogenized, and incubated for 1 h at 4°C in the presence of protease inhibitors (Bioshop, Inc.) and Triton X-100 at a final concentration of 2% (Bioshop, Inc.) prior to lyophilization. Protease inhibitors (Bioshop, Inc.) were added to the culture supernatant, lyophilized, and stored at −20°C. Detection of Agd3-RFP was performed by Western blotting using rat anti-RFP antibody (Chromotek, Inc.) and horseradish peroxidase (HRP)-conjugated donkey anti-rat IgG (Chromotek, Inc.).

For liquid chromatography-mass spectrometry (LC-MS) analysis, bands were excised from sodium dodecyl sulfate-polyacrylamide gels (SDS-PAGs), washed in 0.1 M ammonium bicarbonate, and incubated first in 10 mM dithiothreitol for 45 min at 56°C and then in 0.1 M ammonium bicarbonate containing 55 mM iodoacetamide for 20 min at room temperature. The gel was washed in 0.1 M ammonium bicarbonate−acetonitrile (1:1, vol/vol) for 15 min and incubated in sequencing grade modified trypsin (Promega) for 1 h at 4°C and then overnight at 37°C. Peptides were extracted from the gel by successive incubations of 10 min in acetonitrile, 0.1 M ammonium bicarbonate, acetonitrile, and 5% formic acid. Pooled supernatants were lyophilized, resolubilized in 0.1% aqueous formic acid, and then loaded onto a Thermo Acclaim PepMap precolumn (75-µm inside diameter [i.d.] by 2 cm with 3-µm C_18_ beads) and a Thermo PepMap EASY-spray analytical column (75 µm by 15 cm with 2-µm C_18_ beads) for separation using a Dionex NLC 3000 liquid chromatograph (LC) at 200 nl/min with a gradient of 2 to 35% organic 0.1% formic acid in acetonitrile over 2 h. Peptides were analyzed using a Thermo Orbitrap Fusion mass spectrometer operating at 120,000 resolution (full width at half maximum [FWHM] in the first stage of mass analysis [MS1] and 15,000 for tandem mass spectrometry [MS-MS]) with higher-energy collision-induced dissociation (HCD) sequencing of all peptides with a charge of 2+ or greater. Amino acid sequence was queried using the X! Tandem (Beavis Informatics) search engine VENGEANCE (accessed on 15 December 2015) and analyzed and visualized using Scaffold Q+ Scaffold_4.4.8 (Proteome Sciences). Confirmatory analysis was performed using Pinnacle v1.0.30.0 (Optys Technologies) and the integrated counts from MS1 extracted ion currents specific to peptides in RFP and Agd3 verified by MS-MS.

### GAG enzyme immunoassay (EIA).

Culture filtrates from wild-type, Δ*uge3* mutant, or Δ*agd3* mutant strains alone or in combination were incubated for 1 h in a high-binding enzyme-linked immunosorbent assay (ELISA) plate (Nunclon Inc.). The wells were then washed with washing buffer, incubated for 1 h on a rotator with anti-GAG antibody (a kind gift from Jean-Paul Latgé, Institut Pasteur, Paris, France), then washed, incubated with anti-IgG HRP-tagged secondary antibody (Jackson Laboratories, Inc.), and developed by adding HRP substrate solution (Clontech, Inc.).

### Virulence studies.

Male BALB/c mice, 5 to 6 weeks old, were infected with the fungal strain indicated in the figures or sham infected with phosphate-buffered saline (PBS) using an aerosol chamber as previously described (30). Mice were immunosuppressed with 250 mg of cortisone acetate (Sigma-Aldrich) per kg of body weight by subcutaneous injection on days −2 and +3 and with 250 mg of cyclophosphamide (Western Medical Supply, Inc.) per kg intraperitoneally on day −2 and 200 mg/kg on day +3 relative to conidial challenge (30). To prevent bacterial infection, enrofloxacin was added to the drinking water (Baytril, Inc.). Mice were monitored for a period of 2 weeks for signs of illness, and moribund animals were euthanized.

For pulmonary fungal burden determination, eight 5- to 6-week-old immunosuppressed male BALB/c mice were infected endotracheally with the fungal strains indicated in the figures as previously described (54). At day 4 postinfection, the lungs were harvested and either homogenized for fungal burden determination or fixed in formalin for histopathology. Fungal burden in lung homogenates was quantified by PCR, or the relative content of galactomannan was determined as previously described (2). To assess the extent of lung injury, bronchoalveolar lavage fluid samples from infected animals were collected prior to harvesting the lungs, and the activity of lactose dehydrogenase was determined per the manufacturer’s instructions (CytoTox96 nonradioactive cytotoxicity assay kit; Promega). For pulmonary histopathology examination, sections of lung were stained with periodic acid-Schiff (PAS) and digitally scanned at the Institute for Research in Immunology and Cancer (Université de Montréal). Scanned images were viewed, analyzed, and captured using ObjectiView (Objective Pathology Services). All procedures involving mice were approved by the Los Angeles Biomedical Research Institute Animal Use and Care Committee and the McGill University Animal Use and Care Committee.

## SUPPLEMENTAL MATERIAL

Figure S1  Deletion of *agd3* does not affect susceptibility to caspofungin or nikkomycin. Hyphae of the indicated strains were grown in the presence of caspofungin and nikkomycin. No difference in sensitivity to antifungal agents was observed between the indicated strains, and no morphological differences were observed between hyphae of these strains at subinhibitory concentrations of caspofungin (0.25 µg/ml) or nikkomycin (0.032 µg/ml). The magnification for all images is ×200. Download Figure S1, TIF file, 2 MB

Figure S2  Deletion of *agd3* increase dectin-1 binding on the hyphal surface. The average pixel quantification of fluorescence in Fig. 4D was measured using ImageJ. Download Figure S2, TIF file, 2 MB

Figure S3  Culture filtrates from the Δ*uge3* mutant complement the defects in the production of functional GAG of the Δ*agd3* mutant. Adherent, deacetylated GAG in culture supernatants of the indicated strains was detected by indirect ELISA. Values are means plus standard errors of the means (SEM) (error bars). The values for strains that are significantly different from the value for wild-type *A. fumigatus* Af293 strain (*P* < 0.05 by ANOVA with Tukey’s test for pairwise comparison) are indicated by an asterisk. The values for wild-type *A. fumigatus* and the other Δ*agd3* mutant strain were not statistically significantly different (n.s.). Download Figure S3, TIF file, 1.5 MB

Figure S4  Detection of Agd3 in mycelia and culture supernatants by Western blotting. Culture supernatant or biomass was harvested from cultures of the indicated strains after 24-h growth. Proteins from each fraction were immunoblotted for detection of RFP. Samples obtained from two independent growth and extraction experiments are shown. Lanes: 1, wild-type Af293 culture supernatant; 2, wild-type Af293 biomass; 3, Agd3-RFP strain culture supernatant; 4, Agd3-RFP strain biomass; 5, recombinant RFP; 6, Agd3-RFP strain culture supernatant; 7, Agd3-RFP strain biomass. The positions of molecular weight (MW) markers (in thousands) are indicated to the right of the blot. Download Figure S4, TIF file, 2 MB

Figure S5  Schematic of the GAG biosynthetic cluster in multiple fungi illustrating the degree of synteny of the cluster genes. Each arrow represents a predicted gene product required for GAG synthesis (yellow) or another putative functional or hypothetical protein (blue). A star indicates a small peptide/protein typically less than 100 amino acids with a predicted open reading frame. The direction of the arrow indicates the direction of predicted transcription. Download Figure S5, TIF file, 2.4 MB

Table S1  List of pathogenic species in which the GAG gene cluster was identified. Table S1, XLSX file, 0.04 MB

Table S2  List of primers. Table S2, XLSX file, 0.04 MB
