# Supplementary figures and images for: Deacetylation of Fungal Exopolysaccharide Mediates Adhesion and Biofilm Formation
Source: mBio. 2016 Apr 5;7(2):e00252-16. doi: 10.1128/mBio.00252-16 (PMC4817252; doi:10.1128/mBio.00252-16)

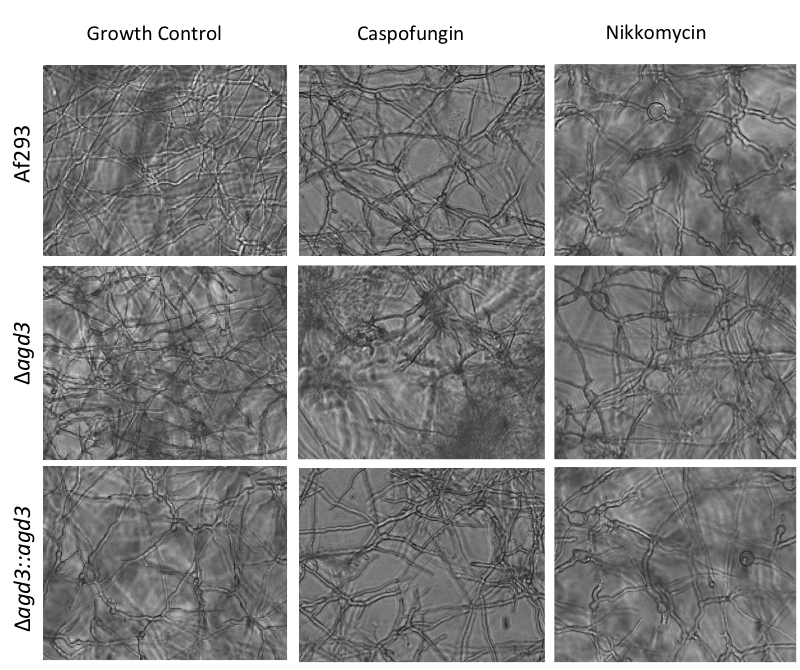

Supplement: Figure S1 — Deletion of agd3 does not affect susceptibility to caspofungin or nikkomycin. Hyphae of the indicated strains were grown in the presence of caspofungin and nikkomycin. No difference in sensitivity to antifungal agents was observed between the indicated strains, and no morphological differences were observed between hyphae of these strains at subinhibitory concentrations of caspofungin (0.25 µg/ml) or nikkomycin (0.032 µg/ml). The magnification for all images is ×200. Download [file mbo002162745sf1.tif]

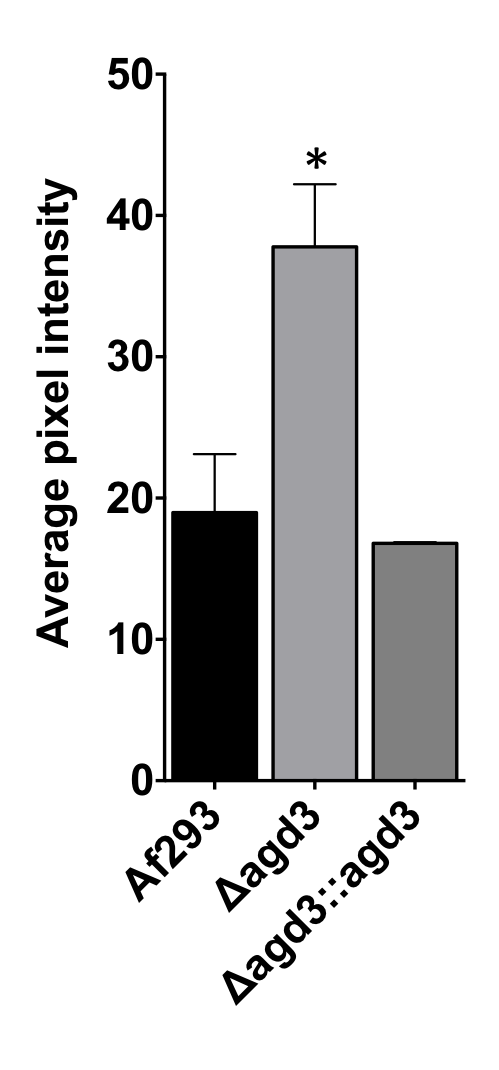

Supplement: Figure S2 — Deletion of agd3 increase dectin-1 binding on the hyphal surface. The average pixel quantification of fluorescence in Fig. 4D was measured using ImageJ. Download [file mbo002162745sf2.tif]

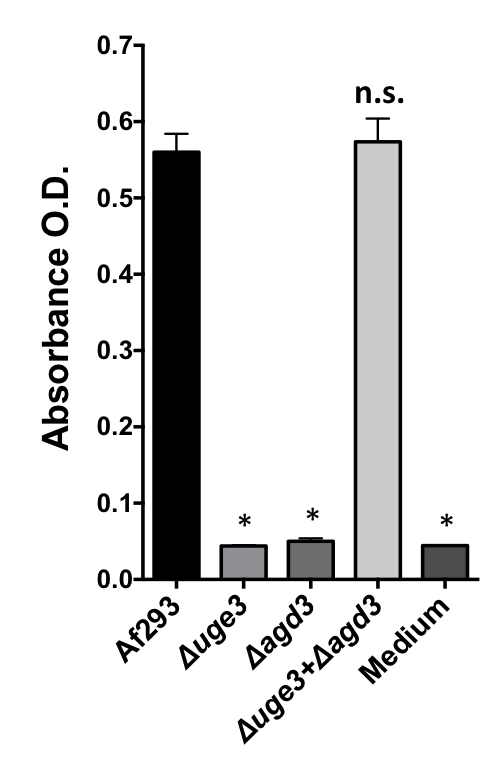

Supplement: Figure S3 — Culture filtrates from the Δuge3 mutant complement the defects in the production of functional GAG of the Δagd3 mutant. Adherent, deacetylated GAG in culture supernatants of the indicated strains was detected by indirect ELISA. Values are means plus standard errors of the means (SEM) (error bars). The values for strains that are significantly different from the value for wild-type A. fumigatus Af293 strain (P < 0.05 by ANOVA with Tukey’s test for pairwise comparison) are indicated by an asterisk. The values for wild-type A. fumigatus and the other Δagd3 mutant strain were not statistically significantly different (n.s.). Download [file mbo002162745sf3.tif]

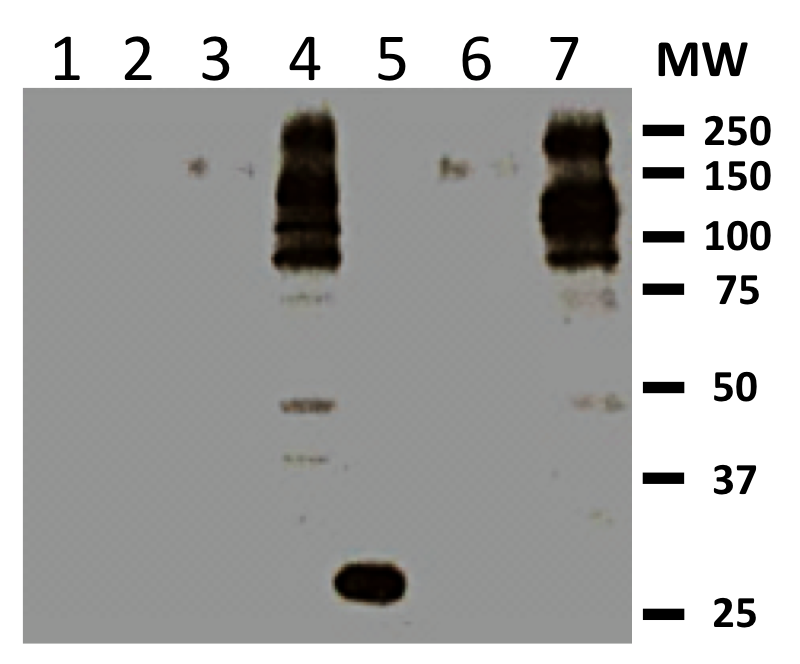

Supplement: Figure S4 — Detection of Agd3 in mycelia and culture supernatants by Western blotting. Culture supernatant or biomass was harvested from cultures of the indicated strains after 24-h growth. Proteins from each fraction were immunoblotted for detection of RFP. Samples obtained from two independent growth and extraction experiments are shown. Lanes: 1, wild-type Af293 culture supernatant; 2, wild-type Af293 biomass; 3, Agd3-RFP strain culture supernatant; 4, Agd3-RFP strain biomass; 5, recombinant RFP; 6, Agd3-RFP strain culture supernatant; 7, Agd3-RFP strain biomass. The positions of molecular weight (MW) markers (in thousands) are indicated to the right of the blot. Download [file mbo002162745sf4.tif]

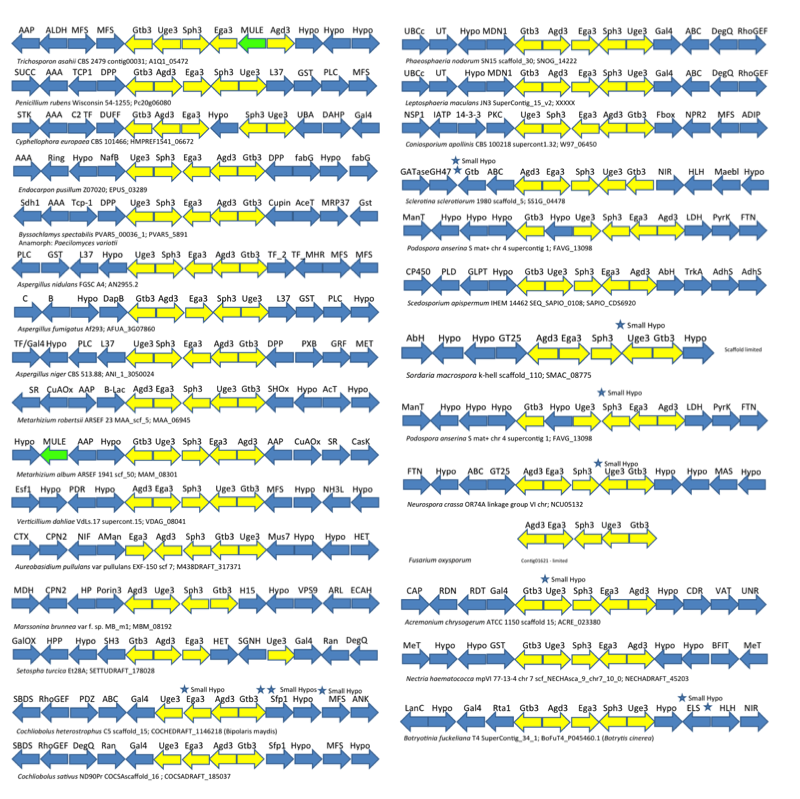

Supplement: Figure S5 — Schematic of the GAG biosynthetic cluster in multiple fungi illustrating the degree of synteny of the cluster genes. Each arrow represents a predicted gene product required for GAG synthesis (yellow) or another putative functional or hypothetical protein (blue). A star indicates a small peptide/protein typically less than 100 amino acids with a predicted open reading frame. The direction of the arrow indicates the direction of predicted transcription. Download [file mbo002162745sf5.tif]
